# Effects of a Motion-Triggered Neuromuscular Electrical Stimulation Strength Program on Shoulder Strength and Throwing Velocity in Elite Handball Players

**DOI:** 10.3390/jcm15041420

**Published:** 2026-02-11

**Authors:** Sebastian Conner-Rilk, Fabian M. Tomanek, Brenda Laky, Philipp R. Heuberer, Jakob E. Schanda, Ulrich Lanz

**Affiliations:** 1 Department of Orthopaedic Surgery, Hospital for Special Surgery, New York-Presbyterian, Weill Medical College of Cornell University, New York, NY 10021, USA; 2 Department of Orthopedics and Trauma Surgery, Medical University of Vienna, 1090 Vienna, Austria; 3 Department for Trauma Surgery, AUVA Trauma Center Vienna-Meidling, 1120 Vienna, Austria; 4 Clinical Research Center, University Clinic of Dentistry, Medical University of Vienna, 1090 Vienna, Austria; 5 Austrian Research Group for Regenerative and Orthopedic Medicine (AURROM), 1050 Vienna, Austria; 6 Austrian Society of Regenerative Medicine, 1010 Vienna, Austria; 7 Medical Faculty, Sigmund Freud University, 1020 Vienna, Austria; 8 healthPi Medical Center, 1010 Vienna, Austria; 9 Ludwig Boltzmann Institute for Traumatology, The Research Center in Cooperation with AUVA, Austrian Cluster for Tissue Regeneration, 1200 Vienna, Austria; 10 Sportorthopädie Zentrum, 1130 Vienna, Austria

**Keywords:** motor control, athletic performance, shoulder instability

## Abstract

**Background:** To evaluate the effects of a motion-triggered neuromuscular electrical stimulation (NMES) shoulder strengthening program on rotational shoulder strength and throwing velocity in healthy, elite-level handball players. **Methods:** Fourteen male handball players were randomly allocated (1:1) to either the NMES or control group. Participants were assessed by a blinded investigator at baseline and after 6 weeks for clinical status, isometric dynamometer-based external (ER) and internal rotational (IR) maximal shoulder strength, and handball endurance and maximal throwing velocity (7 m free throw). Between time points, NMES subjects completed a standardized motion-triggered NMES shoulder strengthening program (3 sessions/week, 30 min for 6 weeks), whereas controls performed a conventional standardized strength program. **Results:** After completion of the motion-triggered NMES program, all NMES participants (100%) demonstrated significant gains in isometric ER strength (+1.4 ± 1.1 kg, *p* = 0.016) compared with 43% of controls, who demonstrated no overall improvement (−0.2 ± 1.8 kg, *p* = 0.740). Similarly, a significantly greater proportion of NMES participants improved endurance throwing velocity compared with controls (100% vs. 29%, *p* = 0.004), with a mean increase of +2.9 ± 2.8 km·h^−1^ (*p* = 0.0.56). Maximum throwing velocity showed no between-group differences in the proportion of athletes with improved results (*p* = 0.899). **Conclusions**: A six-week motion-triggered NMES shoulder strengthening program improved external rotation strength and increased the proportion of athletes demonstrating enhanced endurance throwing velocity under fatigued conditions. However, when compared with conventional exercise alone, NMES did not confer additional benefits for maximal throwing velocity in this study. Therefore, NMES should be regarded as a complementary modality rather than a substitute for established shoulder strengthening exercises.

## 1. Introduction

Handball is characterized by repetitive overhead throwing motions that place high demands on shoulder strength, coordination, and fatigue resistance [1,2,3]. Previous research has highlighted the importance of neuromuscular control and coordinated muscle activation for maintaining shoulder function during repetitive overhead activities [4,5,6]. In this context, motion-triggered neuromuscular electrical stimulation (NMES) has been shown to enhance neuromuscular coordination and shoulder control, providing a mechanistic rationale for its potential application in performance-oriented neuromuscular training [1,2,7].

By enhancing muscular balance and intermuscular coordination, motion-triggered NMES may convert initially non-controllable movement patterns into controllable ones, suggesting potential applications in performance-oriented neuromuscular training [2,7]. Given that the overhead throw is the most important sport-specific motion in handball [3], primarily determined by throwing velocity and precision [8,9], improving these parameters through enhanced intermuscular coordination and balanced muscle strength [8,10], may be feasible using motion-triggered NMES shoulder strengthening.

Therefore, the aim of this study was to evaluate a motion-triggered NMES shoulder strengthening program using objective clinical outcome parameters and to assess its effect on rotational shoulder strength and throwing velocity in healthy, elite-level handball players. It was hypothesized that a 6-week motion-triggered NMES shoulder strengthening program would improve external rotation (ER) shoulder strength and may positively influence throwing velocity by enhancing motor control.

## 2. Materials and Methods

This study was performed in accordance with the Declaration of Helsinki and approved by the Ethics Committee of the City of Vienna (EK 22-201-1022). Written informed consent was obtained from all participants before inclusion. Eligible participants were active elite-level handball players competing in the Austrian first national handball league and participating in a minimum of five structured team-based training sessions per week. Exclusion criteria included (i) age < 18 years, (ii) history of type I or II shoulder instability according to the Stanmore classification [11], (iii) existing shoulder pain syndrome (defined as pain at rest or during motion unrelated to that which impedes training), or (iv) recent shoulder surgery within 1 year. Participants were randomly allocated (1:1 ratio) to either the NMES or control group. Randomization was performed by the team’s head coach, who drew sealed opaque envelopes containing study IDs pre-assigned to either the NMES or control group. The head coach was blinded to group assignment during the draw to minimize selection bias. After allocation, group assignments were revealed to the head coach to allow supervision of the training sessions. All study investigators and authors remained blinded to group allocation until completion of data collection and analysis.

The primary outcome of this randomized prospective trial was the change in throwing velocity (km·h^−1^) from baseline to 6-week post-intervention follow-up. The secondary outcome was the change in isometric ER and internal rotation (IR) maximal shoulder strength measured with a hand-held dynamometer (HHD).

At baseline, participants underwent a standardized clinical and performance assessment. Before the start of the performance testing, participants completed a 5 min standardized warm-up consisting of cardiovascular activation, mobilization, and dynamic stretching exercises. Performance assessment included handball throwing velocity and isometric HHD ER and IR maximal shoulder strength testing. After completion of baseline evaluation and confirmation of health and full performance status, randomized and examiner-blinded group allocation was conducted (NMES (*n* = 7) and control group (*n* = 7)).

Baseline assessment was performed during the preseason transition into the competitive season and repeated at 6 weeks in the same indoor court setting, at the same time of the day (5:00 p.m.), and 2 days after the most recent match.

### 2.1. Clinical Examination

Clinical baseline evaluation was performed to exclude existing shoulder pathology, particularly type I or II shoulder instability [11]. All assessments were performed by a single fellowship-trained orthopedic shoulder specialist (U.L.). Each participant underwent evaluation of range of motion (ROM), repetitive resistance external and internal rotation stress test (RERST, RIRST), Posterior Plier test, O’Brien test as well as Belly Press test. Visual inspection for infraspinatus muscle atrophy and scapular dyskinesis (sick scapula sign) [12] was also performed. Passive ER and IR shoulder ROM were measured with a manual goniometer in the supine position on a bench, with 90° shoulder abduction and 90° elbow flexion. The examiner stabilized the participant’s proximal shoulder region (clavicle and scapula) against the bench while moving the shoulder to maximal passive ER and IR.

### 2.2. Handball Throwing Velocity

**Figure 1:**
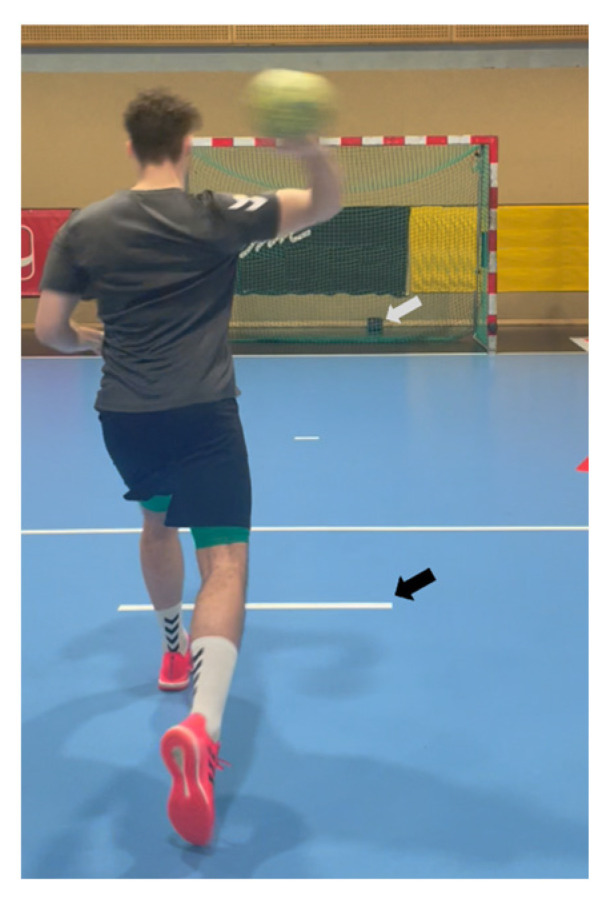
Handball throwing velocity testing setup. A player performing an overhead throw from behind the 7 m free-throw line (black arrow). The radar gun (gray arrow) was positioned behind the net to record the throwing velocity.

Throwing velocity (km·h^−1^) was measured during a standing 7 m throw using a calibrated radar gun positioned behind the net. Radar-based assessment of throwing velocity has demonstrated high validity and reliability in overhead sports (ICC > 0.90) [8,9,13]. Participants were instructed to perform an overhead throw with maximum effort to record top speed from the 7 m free-throw line (Figure 1). After a standardized warm-up, players completed five sub-maximal (50%), three moderate (75%), and two maximal (100%) throws. For testing, five maximal throws were performed with 30 s rest intervals to determine maximal throwing velocity, followed by a 5 min break and ten consecutive throws (≤5 s rest), aiming to reach muscle fatigue and assess endurance throwing velocity. Only throws hitting the net and detected by the radar gun were included. The mean of the five maximal and ten endurance throws was used for analysis.

### 2.3. Isometric HHD ER and IR Maximal Shoulder Strength

**Figure 2:**
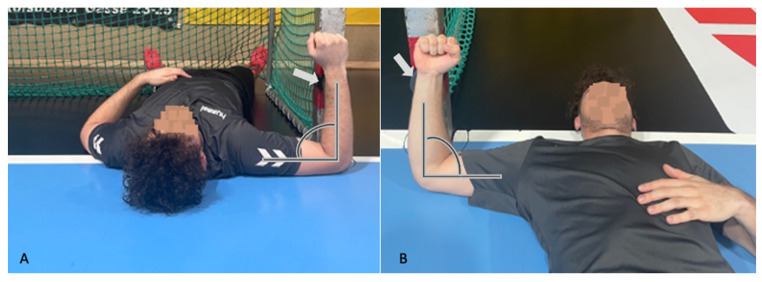
Isometric hand-held dynamometer external and internal rotation maximal shoulder strength testing setup. Participants were positioned supine with 90° shoulder abduction and 90° elbow flexion. The hand-held dynamometer was fixed to the goal post (gray arrow) to measure maximal (**A**) external and (**B**) internal rotational shoulder strength.

Participants were positioned supine with 90° shoulder abduction and 90° elbow flexion. Maximal isometric ER and IR strength were assessed using a calibrated HHD (Activforce 2, Activbody, San Diego, CA, USA) fixed to a goal post to maintain uniform stabilization. Hand-held dynamometry has demonstrated good-to-excellent reliability for assessing isometric shoulder rotational strength in athletic populations (ICC = 0.85–0.98) [7,14]. For ER strength, the HHD was placed proximal to the ulnar styloid; for IR strength, proximal to the radial styloid. (Figure 2A,B). Testing was performed for both the dominant (throwing) and non-dominant shoulders. Each participant performed three 5 s maximal efforts with 30 s of rest between repetitions. The mean force (kg) of three maximal trials was used for final analysis.

### 2.4. Motion-Triggered NMES Shoulder Strengthening Program

Between baseline and 6-week follow-up, the NMES group performed a standardized motion-triggered NMES shoulder strengthening program (3 sessions/week, 30 min per session for 6 weeks) using the Shoulder Pacemaker (NCS Lab Srl, Modena, Italy), an FDA and CE-approved motion-activated stimulation device designed to correct shoulder muscle imbalance. The system adjusts NMES intensity according to the angle of motion of the arm to induce subtetanic contractions and promote supraspinal neural adaptations, as previously described in clinical and experimental studies investigating motion-triggered NMES for shoulder neuromuscular control [15,16,17].

**Figure 3:**
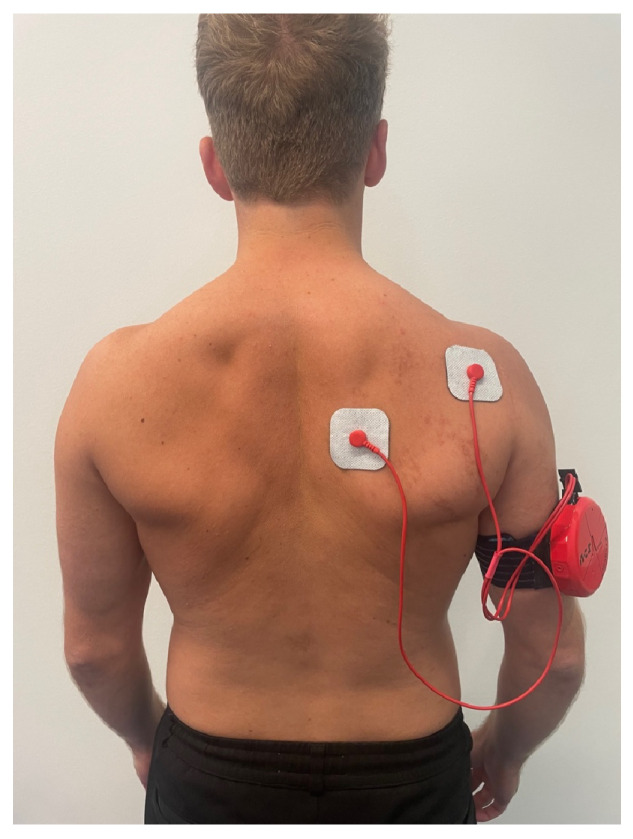
Shoulder pacemaker electrode placement. Electrodes were positioned as specified by the manufacturer: electrode 1, inferior to the spina scapulae (stimulating the infraspinatus, teres minor, and posterior deltoid) and electrode 2, medial to the scapula border (stimulating the lower trapezius and rhomboids).

The training protocol included concentric, eccentric, and functional training (Table 1). Electrodes were placed per manufacturer recommendations: electrode 1, inferior to the scapular spine (stimulation of the infraspinatus, teres minor, and posterior deltoid); and electrode 2, medial to the medial border of the scapula (stimulation of the lower trapezius and rhomboids) (Figure 3).

The control group performed an identical strengthening program using conventional resistance exercises (e.g., elastic bands or body-weight resistance with the same frequency, duration, and movement patterns, but without NMES).

After the 6-week intervention, all participants were re-evaluated by the same blinded examiner (U.L.) using the same evaluation protocol.

### 2.5. Statistical Analysis

Statistical analysis was performed with SPSS Statistics Version 28 (IBM Corporation, Armonk, NY, USA). All statistical tests were two-sided, with significance set at *p* < 0.05. Descriptive statistics summarized participant characteristics. Data distribution was assessed visually using histograms and analytically using the Shapiro–Wilk-test. Normally distributed data are presented as mean ± standard deviation (SD), non-normally distributed data as median and interquartile range (IQR). Categorical variables were compared using Chi-square tests. Independent samples *t*-tests (parametric) or Mann–Whitney U-tests (non-parametric) compared group differences, while paired *t*-tests (parametric) assessed within-group changes from baseline to follow-up.

A post hoc power analysis using G*Power Version 3.1.9.7 indicated that the study had 81% power to detect a mean difference of 3 ± 2.9 km·h^−1^ in throwing velocity between groups at α = 0.05.

## 3. Results

Out of 16 available professional players from an Austrian first league handball team, two players were excluded according to predefined exclusion criteria (age < 18 years, *n* = 1; type I/II shoulder instability, *n* = 1). A total of 14 male handball players (mean age 19.6 ± 1.6 years) were included in the study. Baseline demographic and physical characteristics were comparable between groups (Table 2). A detailed overview of all outcome measures is presented in Table 3 and Table 4.

### 3.1. Handball Throwing Velocity

One participant in the NMES group was unable to complete post-intervention testing due to a non-study-related hamstring injury; therefore, six NMES and seven control participants were included in the final throwing velocity analysis. A detailed overview of all outcome handball throwing velocity measures is presented in Table 3.

### 3.2. Isometric HHD ER and IR Maximal Shoulder Strength

All NMES participants (100%, *n* = 7) demonstrated an increase in isometric ER strength of the dominant shoulder (+1.4 ± 1.1 kg, *p* = 0.016), while the control group showed no gains (−0.2 ± 1.8 kg, *p* = 0.740), with only 43% (*n* = 3) improving (Table 4). No relevant strength changes were observed for the non-dominant shoulder (NMES, +0.9 ± 2.5 kg, *p* = 0.360; control, +0.4 ± 2.9, *p* = 0.728). Isometric IR strength did not differ significantly between groups (*p* = 0.986) or between baseline and follow-up for either group (NMES, *p* = 0.577; control *p* = 0.478).

## 4. Discussion

The principal findings of this study were that a 6-week motion-triggered NMES shoulder strengthening program led to significant gains in isometric ER strength and a trend toward improved endurance throwing velocity in elite-level handball players. Although all NMES participants showed improvements in external rotation strength, these adaptations were not accompanied by superior maximal throwing velocity compared with controls, who demonstrated similar improvements under rested conditions. In contrast, the performance-related effects of the intervention were observed only under fatigued conditions, reflected by improvements in endurance throwing velocity. Collectively, these findings indicate that motion-triggered NMES induces neuromuscular adaptations that preferentially support fatigue-related performance rather than peak throwing speed and therefore may serve as an adjunct to conventional shoulder conditioning rather than a replacement for performance-oriented strength training.

The overhead throw is the key determinant of performance in handball [3], primarily influenced by throwing velocity and precision [8,9]. These parameters depend on optimal intermuscular coordination and balanced muscle strength [8,10]. Numerous studies have investigated the effect of resistance training on throwing velocity in handball players, yet results have been inconsistent [14]. A recent meta-analysis concluded that further high-quality studies are needed due to substantial heterogeneity and low levels of evidence in prior studies [14]. Previous interventional studies reporting positive effects on handball throwing velocity have generally employed dynamic weightlifting, plyometric and heavy resistance training [9,18,19,20,21,22] or medicine ball-based training protocols [13,23]. In contrast to these workload-intensive approaches [9,13,18,19,20,21,22,23], the motion-triggered NMES program is based on a motion activation stimulation technology that detects muscle activity and selectively triggers muscle contractions according to specific movement patterns. This method provides sensory, auditory, and visual biofeedback to enhance motor learning and neuromuscular control.

In this study, the motion-triggered NMES shoulder strengthening program improved endurance throwing velocity, reflecting a fatigued muscle state, whereas maximal throwing velocity under rested conditions remained unchanged. Although maximal throwing speed represents an important performance determinant, throwing velocity in handball is a multifactorial outcome that depends not only on peak force production but also on intermuscular coordination, timing, and balanced shoulder strength. Importantly, fatigue is known to impair force generation and intermuscular coordination, ultimately compromising motor performance during repetitive overhead actions [24,25,26,27]. However, the fatigue protocol used in the present study was designed to induce a standardized and reproducible fatigued state rather than to replicate the complex and intermittent demands of competitive handball match play. In this context, the observed improvement in endurance throwing velocity is consistent with the underlying rationale of the intervention and with prior literature demonstrating that throwing performance under repetitive loading relies heavily on coordinated muscle activation and muscular balance rather than maximal force alone [17,19]. Motion-triggered NMES is specifically designed to enhance neuromuscular control and coordinated activation patterns, which may preferentially support the maintenance of throwing velocity under fatigued conditions. This interpretation is further supported by Moroder et al., demonstrating that motion-triggered NMES-based protocols can enhance muscular coordination and shoulder control by improving neuromuscular activation patterns [1,2,7]. While these neuromuscular adaptations were measurable, they primarily transferred to sustained throwing performance under fatigue and did not confer additional benefits for maximal throwing velocity beyond conventional training, reflecting the multifactorial nature of throwing performance in elite athletes.

### Limitations

This study has several limitations. First, the small sample size inherent to this pilot design limits statistical power and increases susceptibility to type I error, particularly in the presence of multiple outcome measures. Accordingly, the findings should be regarded as exploratory rather than confirmatory. Second, the study population consisted exclusively of male elite-level handball players, which restricts generalizability to female athletes, other performance levels, or different overhead sports. Third, although group allocation was performed using concealed envelopes and investigators remained blinded until completion of data analysis, randomization was not stratified, which may have contributed to baseline differences between groups. Fourth, the control intervention was not matched for biofeedback, novelty, or sensory stimulation associated with NMES; therefore, motivational or placebo effects cannot be fully excluded. Future studies incorporating a sham NMES condition would be required to better isolate physiological effects. Additionally, shoulder strength was assessed using isometric hand-held dynamometry. While this method is reliable and practical, it may not fully reflect dynamic or velocity-dependent strength characteristics relevant to throwing performance. Finally, only short-term outcomes over a six-week period were evaluated; therefore, the persistence of the observed neuromuscular adaptations and their potential long-term performance relevance remain unknown.

## 5. Conclusions

A six-week motion-triggered NMES shoulder strengthening program improved external rotation strength and increased the proportion of athletes demonstrating enhanced endurance throwing velocity under fatigued conditions. However, when compared with conventional exercise alone, NMES did not confer additional benefits for maximal throwing velocity in this study. Therefore, NMES should be regarded as a complementary modality rather than a substitute for established shoulder strengthening exercises.

## Figures and Tables

**Table 1:** Motion-triggered neuromuscular electrical stimulation shoulder strengthening protocol.

	Level 1	Level 2	Level 3
**Sets × repetitions**	3 × 20	3 × 20	3 × 20
**Exercise 1**	Arm supported row	Front raises in 45°	Front raises (thumbs up)
**Exercise 2**	Parallel resistance front raises	Crossbody resistance band raises	Cross body ‘tennis forhand’ swing
**Exercise 3**	Rear dealt fly	Single arm resistance band row	Underhand ‘volleyball serve‘ swing

**Table 2:** Baseline patient demographics.

	NMES Group (*n* = 7)	Control Group (*n* = 7)	*p*-Value
**Age (y)**	19.7 ± 2.3 (18–24)	19.6 ± 0.8 (19–21)	0.878
**Height (m)**	1.85 ± 0.06 (1.77–1.93)	1.86 ± 0.04 (1.81–1.91)	0.677
**Weight (kg)**	84.0 ± 17.5 (64.0–120.0)	91.4 ± 10.6 (82.0–120.0)	0.356
**BMI**	21.1 ± 10.1 (20.4–32.2)	26.4 ± 2.8 (23.5–32.0)	0.210
**Arm span (m)**	1.91 ± 6.4 (1.85–2.02)	1.90 ± 5.6 (1.82–1.96)	0.879

Data are presented as mean ± standard deviation (range). BMI, body mass index; NMES, neuromuscular electrical stimulation; y, years.

**Table 3:** Throwing velocity.

		NMES Group (*n* = 6 ^§^)	Control Group (*n* = 7)	Difference	*p*-Value ^1^
**Endurance throwing velocity (km × h^−1^)**	Baseline	87.6 ± 4.9	92.1 ± 3.7	4.4 ± 2.4	0.089
	6-week follow-up	90.5 ± 6.8	93.0 ± 4.5	2.5 ± 3.2	0.448
	Difference	2.9 ± 2.8	0.9 ± 2.9	2.0 ± 1.6	0.244
	*p*-value ^2^	0.056	0.440	-	-
	Improved, *n* (%)	6 (100%)	2 (29%)	-	**0.004**
**Maximum throwing velocity (km × h^−1^)**	Baseline	89.3 ± 6.2	92.4 ± 3.2	3.1 ± 2.7	0.289
	6-week follow-up	92.8 ± 7.5	97.5 ± 4.5	4.7 ± 3.4	0.190
	Difference	3.6 ± 1.7	5.1 ± 4.0	1.6 ± 1.7	0.387
	*p*-value ^2^	**0.004**	**0.014**	-	-
	Improved, *n* (%)	6 (100%)	7 (100%)	-	0.899

^§^ One participant was not able to perform final throwing velocity testing due to a sustained hamstring injury within the study period. Data are presented as mean ± standard deviation. ^1^ *p*-values between study groups. ^2^ *p*-values from baseline to 6-week follow-up. Significant data are presented in bold. NMES, neuromuscular electrical stimulation.

**Table 4:** External and internal rotation shoulder strength.

		NMES Group (*n* = 7)	Control Group (*n* = 7)	Difference	*p*-Value ^1^
**ER strength at 90° (kg)**	Baseline	18.2 ± 3.7	22.9 ± 3.3	4.7 ± 1.9	**0.029**
	Follow-up	19.6 ± 3.7	22.6 ± 3.5	3.0 ± 1.9	0.142
	Difference	1.4 ± 1.1	−0.2 ± 1.8	1.6 ± 0.8	0.061
	*p*-value ^2^	**0.016**	0.740	-	-
	Improved, *n* (%)	7 (100%)	3 (43%)	-	**0.015**
**IR strength at 90° (kg)**	Baseline	18.7 ± 5.5	21.2 ± 6.0	2.5 ± 3.1	0.437
	Follow-up	19.9 ± 4.1	22.4 ± 1.7	2.5 ± 2.1	0.249
	Difference	1.17 ± 5.3	1.2 ± 4.3	0.04 ± 2.6	0.986
	*p*-value ^2^	0.577	0.478	-	-
	Improved, *n* (%)	4 (57%)	4 (57%)	-	*p* > 0.999

Data are presented as mean ± standard deviation. ^1^ *p*-values between study groups. ^2^ *p*-values from baseline to 6-week follow-up. Significant data are presented in bold.

## Data Availability

The data of the findings of this study are available from the “Sportorthopädie Zentrum Hietzing”; however, restrictions apply due to patient confidentiality and further regulations. Therefore, the data is not publicly available. However, data can be provided upon reasonable request and prior approval from the ethical committee.

## Abbreviations

NMES	Neuromuscular electrical stimulation
ER	External Rotation
FPSI	Functional posterior shoulder instability
HHD	Hand-held dynamometer
IR	Internal rotation
ROM	Range of motion
RERST	Repetitive external rotation stress test
RIRST	Repetitive internal rotation stress test
SD	Standard deviation
BMI	Body mass index
Kg	Kilogram
m	Meter
y	Years

## References

[1] Moroder P., Minkus M., Bohm E., Danzinger V., Gerhardt C., Scheibel M. (2017). Use of shoulder pacemaker for treatment of functional shoulder instability: Proof of concept. Obere Extrem..

[2] Moroder P., Plachel F., Van-Vliet H., Adamczewski C., Danzinger V. (2020). Shoulder-Pacemaker Treatment Concept for Posterior Positional Functional Shoulder Instability: A Prospective Clinical Trial. Am. J. Sports Med..

[3] Manchado C., Tortosa-Martinez J., Vila H., Ferragut C., Platen P. (2013). Performance factors in women’s team handball: Physical and physiological aspects--a review. J. Strength Cond. Res..

[4] Meredith S.J., Rauer T., Chmielewski T.L., Fink C., Diermeier T., Rothrauff B.B., Svantesson E., Hamrin Senorski E., Hewett T.E., Sherman S.L. (2020). Return to sport after anterior cruciate ligament injury: Panther Symposium ACL Injury Return to Sport Consensus Group. Knee Surg. Sports Traumatol. Arthrosc..

[5] Aasheim C., Stavenes H., Andersson S.H., Engbretsen L., Clarsen B. (2018). Prevalence and burden of overuse injuries in elite junior handball. BMJ Open Sport Exerc. Med..

[6] Barden J.M., Balyk R., Raso V.J., Moreau M., Bagnall K. (2005). Atypical shoulder muscle activation in multidirectional instability. Clin. Neurophysiol..

[7] Moroder P., Karpinski K., Akgun D., Danzinger V., Gerhardt C., Patzer T., Tauber M., Wellmann M., Scheibel M., Boileau P. (2023). Neuromuscular Electrical Stimulation-Enhanced Physical Therapist Intervention for Functional Posterior Shoulder Instability (Type B1): A Multicenter Randomized Controlled Trial. Phys. Ther..

[8] Marques M.C., van den Tilaar R., Vescovi J.D., Gonzalez-Badillo J.J. (2007). Relationship between throwing velocity, muscle power, and bar velocity during bench press in elite handball players. Int. J. Sports Physiol. Perform..

[9] Hermassi S., Chelly M.S., Fathloun M., Shephard R.J. (2010). The effect of heavy- vs. moderate-load training on the development of strength, power, and throwing ball velocity in male handball players. J. Strength Cond. Res..

[10] Gorostiaga E.M., Granados C., Ibanez J., Gonzalez-Badillo J.J., Izquierdo M. (2006). Effects of an entire season on physical fitness changes in elite male handball players. Med. Sci. Sports Exerc..

[11] Jaggi A., Lambert S. (2010). Rehabilitation for shoulder instability. Br. J. Sports Med..

[12] Burkhart S.S., Morgan C.D., Kibler W.B. (2003). The disabled throwing shoulder: Spectrum of pathology Part III: The SICK scapula, scapular dyskinesis, the kinetic chain, and rehabilitation. Arthroscopy.

[13] Raeder C., Fernandez-Fernandez J., Ferrauti A. (2015). Effects of Six Weeks of Medicine Ball Training on Throwing Velocity, Throwing Precision, and Isokinetic Strength of Shoulder Rotators in Female Handball Players. J. Strength Cond. Res..

[14] Bragazzi N.L., Rouissi M., Hermassi S., Chamari K. (2020). Resistance Training and Handball Players’ Isokinetic, Isometric and Maximal Strength, Muscle Power and Throwing Ball Velocity: A Systematic Review and Meta-Analysis. Int. J. Environ. Res. Public Health.

[15] Vitry F., Martin A., Papaiordanidou M. (2019). Torque gains and neural adaptations following low-intensity motor nerve electrical stimulation training. J. Appl. Physiol..

[16] Kim H., Sandercock T.G., Heckman C.J. (2015). An action potential-driven model of soleus muscle activation dynamics for locomotor-like movements. J. Neural Eng..

[17] Matos F., Amaral J., Martinez E., Canario-Lemos R., Moreira T., Cavalcante J., Peixoto R., Pinheiro B.N., Junior L.S., Uchoa P. (2022). Changes in Muscle Thickness after 8 Weeks of Strength Training, Electromyostimulation, and Both Combined in Healthy Young Adults. Int. J. Environ. Res. Public Health.

[18] Chelly M.S., Hermassi S., Aouadi R., Shephard R.J. (2014). Effects of 8-week in-season plyometric training on upper and lower limb performance of elite adolescent handball players. J. Strength Cond. Res..

[19] Hermassi S., Chelly M.S., Tabka Z., Shephard R.J., Chamari K. (2011). Effects of 8-week in-season upper and lower limb heavy resistance training on the peak power, throwing velocity, and sprint performance of elite male handball players. J. Strength Cond. Res..

[20] Gorostiaga E.M., Izquierdo M., Iturralde P., Ruesta M., Ibanez J. (1999). Effects of heavy resistance training on maximal and explosive force production, endurance and serum hormones in adolescent handball players. Eur. J. Appl. Physiol. Occup. Physiol..

[21] van den Tillaar R. (2004). Effect of different training programs on the velocity of overarm throwing: A brief review. J. Strength Cond. Res..

[22] Hermassi S., Chelly M.S., Bragazzi N.L., Shephard R.J., Schwesig R. (2019). In-Season Weightlifting Training Exercise in Healthy Male Handball Players: Effects on Body Composition, Muscle Volume, Maximal Strength, and Ball-Throwing Velocity. Int. J. Environ. Res. Public Health.

[23] Hermassi S., van den Tillaar R., Khlifa R., Chelly M.S., Chamari K. (2015). Comparison of In-Season-Specific Resistance vs. A Regular Throwing Training Program on Throwing Velocity, Anthropometry, and Power Performance in Elite Handball Players. J. Strength Cond. Res..

[24] Enoka R.M., Stuart D.G. (1992). Neurobiology of muscle fatigue. J. Appl. Physiol..

[25] Aymard C., Katz R., Lafitte C., Le Bozec S., Penicaud A. (1995). Changes in reciprocal and transjoint inhibition induced by muscle fatigue in man. Exp. Brain Res..

[26] Enoka R.M. (1995). Mechanisms of muscle fatigue: Central factors and task dependency. J. Electromyogr. Kinesiol..

[27] De Luca C.J. (1984). Myoelectrical manifestations of localized muscular fatigue in humans. Crit. Rev. Biomed. Eng..

